# Supplementary material for: Dynamic anti-correlations of water hydrogen bonds
Source: Nat Commun. 2024 Dec 1;15:10453. doi: 10.1038/s41467-024-54804-y (PMC11609289; doi:10.1038/s41467-024-54804-y)
Supplement: Supplementary file 1 — Supplementary Information [file 41467_2024_54804_MOESM1_ESM.pdf]

SUPPLEMENTARY INFORMATION

FOR

DYNAMIC ANTI-CORRELATIONS OF WATER HYDROGEN BONDS

Lucas Gunkel[1]†, Amelie A. Ehrhard[1]†, Carola S. Krevert[1], Bogdan A. Marekha[1]‡, Mischa Bonn[1], Maksim Grechko[1], Johannes Hunger[1]*

[1] Max-Planck Institute for Polymer Research, Ackermannweg 10, 55128 Mainz, Germany

† These authors contributed equally to this work

‡ present address: ENSL, CNRS, Laboratoire de Chimie UMR 5182, 46 allée d'Italie, 69364 Lyon France

**Corresponding author. Email: hunger@mpip-mainz.mpg.de.*

Table of contents

Supplementary Notes 1 to 9

Supplementary Figures 1 to 10

Supplementary Tables 1 to 7

Supplementary References

SUPPLEMENTARY NOTES

Supplementary Note 1: Modelling of the linear IR absorption spectra of D_2O in DMF

To estimate the linewidths of the vibrational bands in the IR absorption spectra, we modelled the spectra of D_2O in DMF by a sum of three Gaussian bands to account for the overtone of the D_2O bending vibration, the symmetric O-D stretching band, and the asymmetric O-D stretching mode. The spectra of HOD in DMF were modelled with a single Gaussian band. The experimental spectra and the fits are displayed in Supplementary Figure 1 and the fit parameters are listed in Supplementary Table 1.

Supplementary Note 2: Lorentz and Voigt modelling of the 2D-IR slices

To model the antidiagonal cuts (Figure 2d and 2e in the main manuscript) and determine the homogeneous linewidths, we use a combination of two Lorentzian bands, $l(\tilde{\nu})$:

$$l(\tilde{\nu}) = A_{\mathrm{ESA},i} \cdot \frac{1}{\left(\tilde{\nu}-\tilde{\nu}_{\mathrm{c,ESA},i}\right)^2+\left(\frac{\Gamma_{\mathrm{L,ESA},i}}{2}\right)^2} + A_{\mathrm{GSB},i} \cdot \frac{1}{(\tilde{\nu}-\tilde{\nu}_{\mathrm{c,GSB},i})^2+\left(\frac{\Gamma_{\mathrm{L,GSB},i}}{2}\right)^2} \quad (1)$$

with $A_{\mathrm{ESA},i}$ and $A_{\mathrm{GSB},i}$ (with *i* = *l*, *sym*, *as* for local, symmetric, and asymmetric stretching mode) the amplitudes of the bleaching signal and the excited state absorption, respectively. $\tilde{\nu}_{\mathrm{c,ESA},i}$ and $\tilde{\nu}_{\mathrm{c,GSB},i}$ are the resonance frequencies of the ground state bleaching signal and of the excited state absorption, respectively. To reduce the parameter space, we assume the full widths at half maximum $\Gamma_{\mathrm{L,ESA},i}$ and $\Gamma_{\mathrm{L,GSB},i}$, of both signals to be the same. This assumption cannot be made for the symmetric stretching mode of D_2O since the ESA peak here contains contributions of the symmetric stretching mode and the cross-peak. Therefore, in this case $\Gamma_{\mathrm{L,ESA,s}}$ and $\Gamma_{\mathrm{L,GSB,s}}$ are both adjustable parameters.

To model the diagonal cuts, we fit a Voigt profile, $v(\tilde{\nu})$ – a convolution of a Gaussian profile f_2 representing the purely inhomogeneous distribution with a Lorentz band f_1 – to the data with $\Gamma_{\mathrm{L,GSB},i}$ constrained to the value obtained from fitting Supplementary Eq. 1 to the antidiagonal cuts.

$$v(\tilde{\nu}) = (f_1 * f_2)(\tilde{\nu}) \quad (2)$$

where:

$$f_1(\tilde{\nu}) = \frac{A_i}{2\pi} \frac{\Gamma_{\mathrm{L,GSB},i}}{(\tilde{\nu}-\tilde{\nu}_{\mathrm{c}})^2+\left(\frac{\Gamma_{\mathrm{L,GSB},i}}{2}\right)^2}, f_2(\tilde{\nu}) = \sqrt{\frac{4ln2}{\pi}} \frac{e^{-\frac{4ln2}{\Gamma_{\mathrm{G},i}^2}\tilde{\nu}^2}}{\Gamma_{\mathrm{G},i}} \quad (3)$$

with the amplitude of the Lorentz component A_i and $\Gamma_{G,i}$ the purely inhomogeneous width of the Gaussian distribution. These fits are shown in Figure 2 of the main manuscript and the obtained parameters are listed in Supplementary Tables 2-5.

Supplementary Note 3: Population dynamics

The homogeneous broadening discussed above and in the main manuscript has contributions from population lifetime:[1] longer-lived vibrations give rise to narrower bands. Additionally, accelerated population relaxations for certain modes may indicate different

relaxation pathways, such as Fermi resonances.[2,3] Thus, we quantify the population relaxation via the temporal evolution of the on-diagonal bleaching signals of the different vibrational modes as function of waiting time T_w. We model the integrated 2D-IR signals assuming that the contribution of the excited state decays with a relaxation time τ_i and that the dissipation of the vibrational energy in the course of the relaxation leads to a small and persistent modulation of the oscillator (transient signal due to heat):[4]

$$V(T_\mathrm{w}) = V_\mathrm{exc} \cdot e^{\frac{-T_\mathrm{w}}{\tau_i}} + V_\mathrm{heat} \cdot (1 - e^{\frac{-T_\mathrm{w}}{\tau_i}}) \tag{4}$$

with V_exc and V_heat the magnitude of the contributions of the excitation and the heat, respectively. We integrate the peaks in the shaded areas shown in Supplementary Figure 2. We use elliptical integration areas and the tilt of the ellipse is taken from the tilt of the CLS at $T_w = 100\mathrm{fs}$. Supplementary Figure 3 shows the resulting fits and the thus obtained lifetimes are listed in Supplementary Table 6.

For water we find similar lifetimes for the symmetric and the asymmetric stretching mode. The local mode of HOD molecules shows an increased lifetime compared to the coupled modes. Together with the homogeneous linewidth of the local mode being broader than the linewidths of the coupled mode, these data suggest that population relaxation is not the predominant broadening mechanism.[1,5] For urea we find similar lifetimes for symmetric stretch and asymmetric stretch and the local mode has almost half the lifetime compared to the coupled modes, consistent with the broader homogeneous linewidth of the local mode compared to the coupled stretching modes. The peak volumes of the cross-peaks relax with timescales (see Supplementary Figure 3) similar to the diagonal signals suggesting that the cross-peaks are due to coupling, rather than due to energy transfer or chemical exchange for both D_2O and urea-d4.

Supplementary Note 4: Local correlation maps

For visualization of frequency-frequency correlations in the 2D-IR spectra, we calculate the local, signal-weighted Pearson correlation coefficient r. Therefore, we take an array of 5x5 pixels of the 2D-IR spectra, resulting in 25 data triples $(\tilde{\nu}_\mathrm{probe}; \tilde{\nu}_\mathrm{pump}; |S_\perp(\tilde{\nu}_\mathrm{probe}, \tilde{\nu}_\mathrm{pump})|)$. The correlation coefficient, r, for the center pixel of the 5x5 array is then determined as:

$$r = \frac{cov(\tilde{\nu}_\mathrm{probe}, \tilde{\nu}_\mathrm{pump}, |S_\perp(\tilde{\nu}_\mathrm{probe}, \tilde{\nu}_\mathrm{pump})|)}{\sqrt{cov(\tilde{\nu}_\mathrm{probe}, \tilde{\nu}_\mathrm{probe}, |S_\perp(\tilde{\nu}_\mathrm{probe}, \tilde{\nu}_\mathrm{pump})|) \cdot cov(\tilde{\nu}_\mathrm{pump}, \tilde{\nu}_\mathrm{pump}, |S_\perp(\tilde{\nu}_\mathrm{probe}, \tilde{\nu}_\mathrm{pump})|)}} \tag{5}$$

with $cov(x, y, w) = \frac{\sum_{i=1\cdots25} (x_i - \overline{x}) \cdot (y_i - \overline{y})\, w_i}{\sum_{i=1\cdots25} w_i}$, $\overline{x} = \frac{\sum_{i=1\cdots25} x_i\, w_i}{\sum_{i=1\cdots25} w_i}$, and $\overline{y} = \frac{\sum_{i=1\cdots25} y_i\, w_i}{\sum_{i=1\cdots25} w_i}$. To visualize correlated ($r > 0$), anti-correlated ($r < 0$), or uncorrelated ($r = 0$) spectral regions, we display these coefficients in Figures 4a,b & 5d of the main manuscript and in Supplementary Figure 4, where the signals are smaller than 25% of the minimum signal or greater than 15% of the maximum signal.

Supplementary Note 5: CLS fitting of cross-peak

To determine the centerline position we fitted a Gauss profile to the bleaching or cross peak signal to slices parallel to the probe axis and the center of the Gaussian is taken as the center position. The CLS value is determined from a linear fit of these center points. We model the CLS dynamics of the cross-peak with a combination of a dampened oscillation and an exponential decay:

$$CLS(T_\mathrm{w}) = A_\mathrm{osc} \cdot \sin\left(\left(\frac{T_\mathrm{w}}{\tau_\mathrm{osc}} + \phi\right) \cdot 2\pi\right) \cdot \exp\left(-\frac{T_\mathrm{w}}{\tau_\mathrm{damp}}\right) + A_\mathrm{ex} \cdot \exp\left(-\frac{T_\mathrm{w}}{\tau_\mathrm{ex}}\right) + CLS_\mathrm{offset} \quad (6)$$

with the amplitude of the oscillation A_osc, the oscillation period τ_osc, a phase shift ϕ, the damping constant τ_damp, the amplitude of the exponential decay A_ex, the lifetime of the exponential τ_{ex}, and the CLS offset CLS_offset. This fit is shown in Figure 4c of the main manuscript and the parameters are listed in Supplementary Table 7.

Supplementary Note 6: Estimation of hydrogen bond stretching mode

Liquid water shows broad absorption bands in the THz region at $50\ \mathrm{cm}^{-1}$ (hydrogen-bond bend), $200\ \mathrm{cm}^{-1}$ (hydrogen-bond stretch), and $650\ \mathrm{cm}^{-1}$ (librations).[6] For D_2O the libration band is red-shifted to 530 cm^{-1}.[7] Assuming the potential for the DMF-D_2O hydrogen-bond is the same as for the H_2O-H_2O hydrogen-bonds in neat water, the frequency of the DMF-D_2O hydrogen bond stretch can be estimated from the reduced mass ratio to:

$$200\ \mathrm{cm}^{-1} \cdot \frac{\sqrt{\frac{m_{H_2O} \cdot m_{\mathrm{H_2O}}}{m_{\mathrm{H_2O}} + m_{\mathrm{H_2O}}}}}{\sqrt{\frac{m_{\mathrm{D_2O}} \cdot m_{\mathrm{DMF}}}{m_{\mathrm{D_2O}} + m_{\mathrm{DMF}}}}} \approx 151\ \mathrm{cm}^{-1} \quad (7)$$

which corresponds to an oscillation period of ~220 fs.

Supplementary Note 7: CLS analysis of the diagonal peaks

The CLS of the diagonal peaks show a similar oscillatory pattern as the cross-peak. Moreover, the CLS of the diagonal peaks are reminiscent of what has been reported for H_2O in acetonitrile,[8] for which the CLS decayed on the 50 fs timescale as well as a recurrence at ~200 fs, attributed to underdamped low frequency intermolecular H-bond stretching.[8] The CLS dynamics of the diagonal peaks of the present study can be described with the same oscillatory and fast decay dynamics as the cross-peak, with and additional exponential decay (solid lines in Supplementary Figure 5), with the parameters τ_osc , τ_{damp}, and τ_{ex} constrained to the values obtained from the cross-peak dynamics (Supplementary Table 7). As such, also frequency-frequency correlations for the individual vibrational modes are modulated by the characteristic timescales of water libration and hydrogen bond stretching.

Supplementary Note 8: Fit stability of population fits

In Supplementary Figure 7 we show the numerical fit stability of the fits described in the main manuscript to obtain the H-bond conformation distributions in Figure 4d & 5c. Using a discretized 15x15 (D_2O) and 12x12 (urea) distribution of H-bond configurations $P(d_1, d_2)$, we calculate from these distributions the inhomogeneous distribution of frequencies (Figure 4e & Supplementary Figure 7c) using the frequency maps shown in Figure 3a and Supplementary Figure 11a. We optimize the $P(d_1, d_2)$ distributions by minimizing the deviations of the calculated inhomogeneous distributions from the experimentally determined inhomogeneous distributions (Supplementary Tables S3 & S5). We note that we thereby assume transition dipoles to be independent of frequency. Due to the large parameter space (225 parameters for D_2O and 144 parameters for urea) and due to numerical discretization errors of the frequency

maps, the resulting $P(d_1, d_2)$ distributions are not uniquely determined. To estimate the numerical accuracy, we perform these fits ten times, seeded with random $P(d_1, d_2)$ values. In the main manuscript we show the average distributions obtained from these 10 fits (Figure 4d & 5c). The standard deviations within these 10 fits are displayed in Supplementary Figures 7a&b. Comparison of the data in Supplementary Figures 7a&b to the distributions in Figure 4d & 5c shows that the obtained distributions can be determined sufficiently well to confirm the anticorrelated distributions.

Supplementary Note 9: Linear absorption spectra of urea

Supplementary Figure 9 shows solvent subtracted linear absorption spectra of urea in DMSO with varying isotopic composition at ND stretching frequencies. In the linear absorption spectrum for urea-d4 three peaks are present, the symmetric stretching vibration (*sym*) at 2420 cm^{-1}, the asymmetric stretching vibration (*as*) at 2560 cm^{-1}, and an additional mode at 2360 cm^{-1}. The 2360 cm^{-1} band has also been shown to be present in the solid state.[9] For urea-h4 in the solid state a band adjacent to the N-H stretching band at 3264 cm^{-1} has been ascribed to a combination band of the symmetrical NH_2 deformation and the CO stretching vibration.[10] However, at N-D stretching frequencies a hypothetical combination band of the ND_2 deformation and CO stretching band would be expected at >2600 cm^{-1} and can therefore not explain the 2360 cm^{-1} peak.[9] A contribution of ND_2 deformation modes is even less likely since there seems to be an approximate linear dependence of the peak intensity on the urea-d4 concentration, which is expected for ND stretching but not for ND_2 deformation modes. The linear dependence of the amplitude of this mode on the N-D concentration (Supplementary Figure 9) points to a Fermi resonance including a ND stretching vibration, which in turn leads to a distortion of the line shape of the symmetric stretching mode at 2420 cm^{-1}. Due to this distortion we do not consider the *sym* mode in the analysis in the main manuscript.

In Supplementary Figure 10 we show the IR spectrum of urea-d4/urea (50% / 50%) in DMSO measured over a time period of 2 weeks after preparation. The solution was stored in a glovebox over this time period to avoid uptake of moisture during NH/ND exchange. We find a marked change of the IR spectrum during the first day (different shades of grey). The measured spectrum after two weeks (blue dashed line) is nearly identical to the spectrum after 1 day (red line). We conclude that the NH/ND exchange has reached equilibrium after 1 day. Therefore, all samples measured in this study were prepared at least one day prior to the experiment. The peak at ~2360 cm^{-1} decreases with isotope exchange. As during exchange the amount of fully deuterated urea decreases, this observation supports the assignment of the 2360 cm^{-1} peak to a Fermi resonance.

SUPPLEMENTARY FIGURES

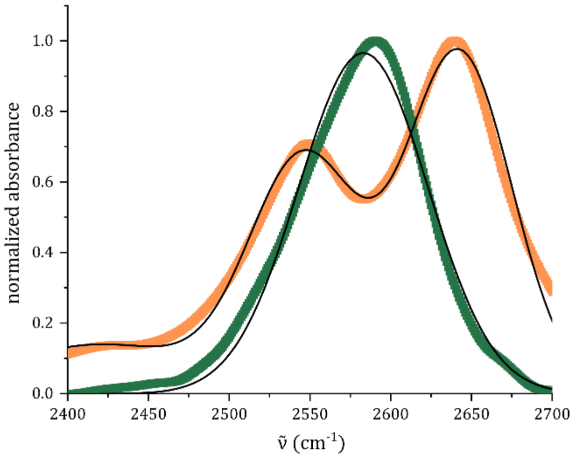

Supplementary Figure 1. Normalized IR spectra of D_2O (orange symbols) and HOD (green symbols) in DMF with Gaussian fits (solid black lines).

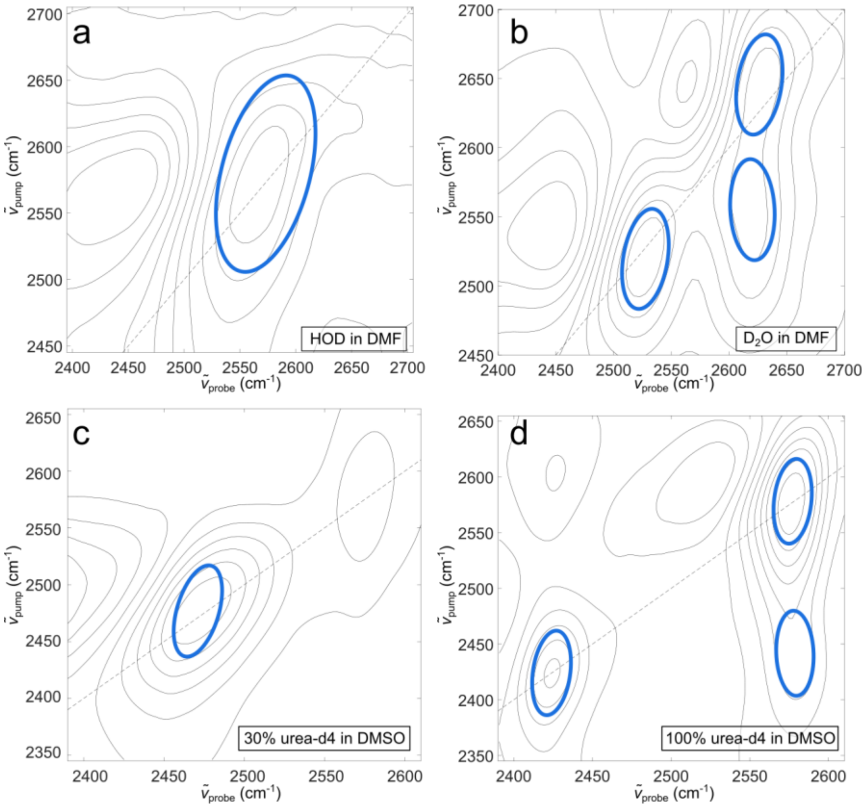

Supplementary Figure 2. Isotropic 2D IR spectra at 100 fs of a) HOD in DMF, b) D_2O in DMF, c) 30% urea-d4 in DMSO and 100 % urea-d4 in DMSO with blue ellipses indicating the integration area for peak volume fitting in Supplementary Figure 3. Integration ellipses are rotated according to the tilt of the CLS at T_w = 100fs.

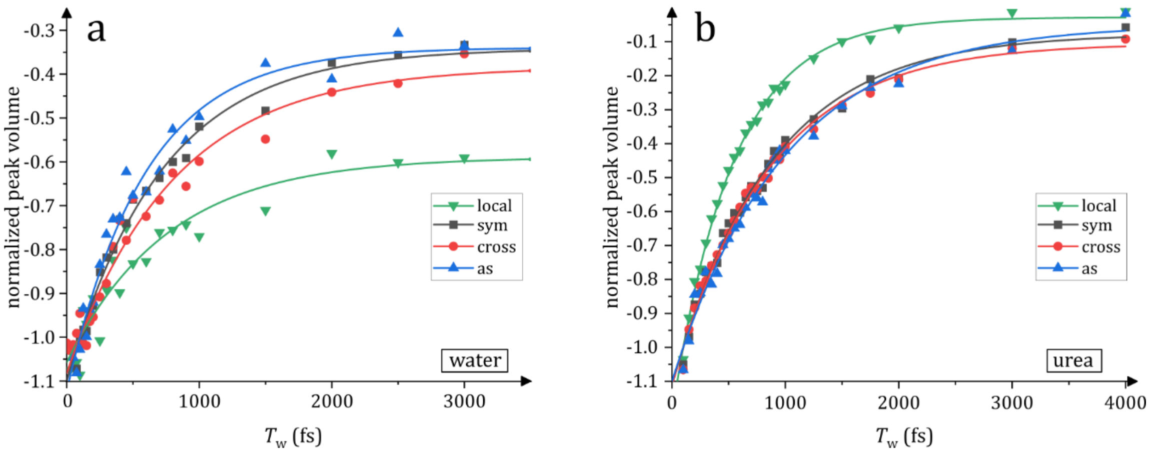

Supplementary Figure 3. Normalized peak volumes of a) water and b) urea with the local mode (green), symmetric stretching (black), cross-peak (red), and asymmetric stretching (blue). Symbols show experimental data and solid lines show fits of Supplementary Eq. 4 to the data.

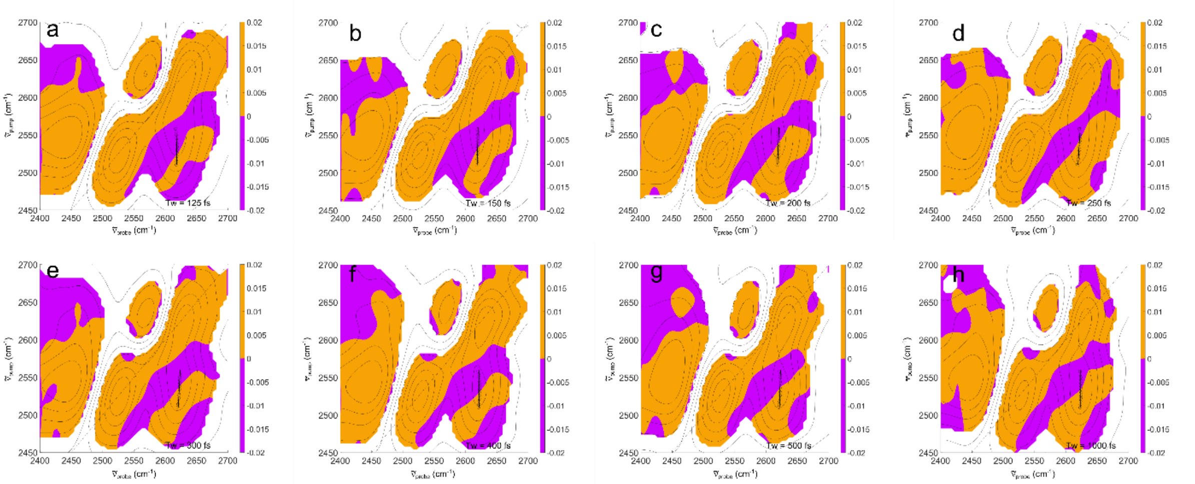

Supplementary Figure 4. Waiting time dependent local correlation maps for D_2O for the perpendicular (<XXZZ>) 2D-IR spectra. a) 125 fs, b) 150 fs, c) 200 fs d) 250 fs, e) 300 fs, f) 400 fs, g) 500 fs and h) 1000 fs. Contour lines show signal intensities, orange and purple areas indicate spectral regions with positive and negative local Pearson correlation coefficients, respectively. Correlation coefficients are only shown where the signals are smaller than 25% of the minimum signal or greater than 15% of the maximum signal. Open blue symbols indicate the center position of the off-diagonal peak and the solid black line the center line.

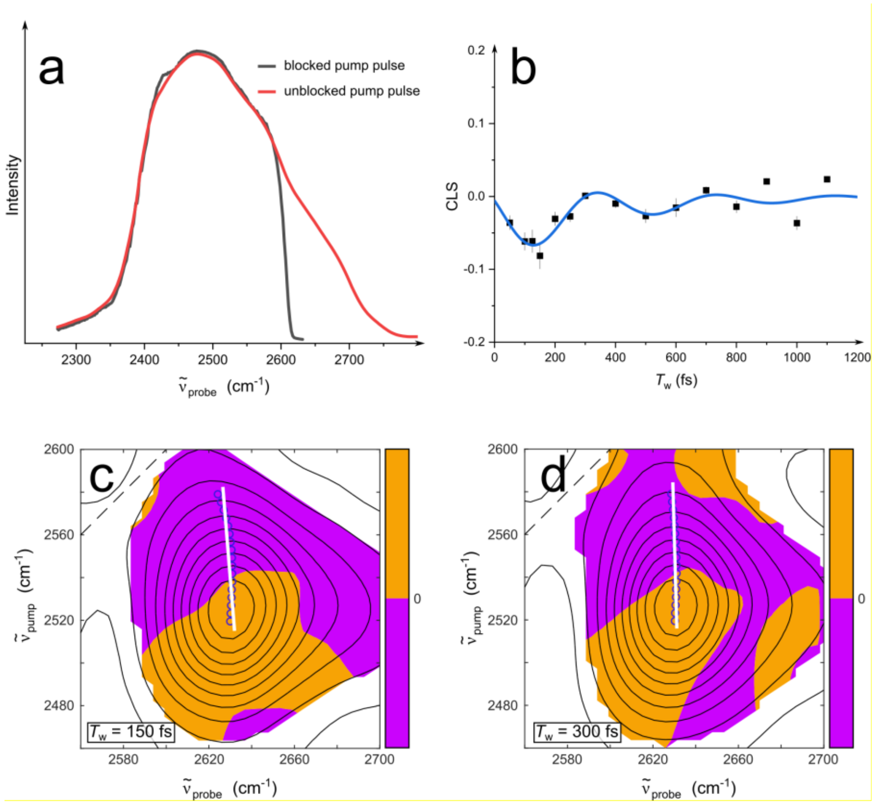

Supplementary Figure 5. a) Pump pulse with high frequencies (> 2625 cm^{-1}) blocked. b) CLS of the crosspeak for D_2O in DMF using the high frequency blocked pump pulse in a). The error bars are the standard error of the linear regression of the center line. The CLS show the same oscillatory pattern as in the main text Figure 4c. This supports the notion that the oscillatory pattern in fact does not stem from coherence transfer between symmetric and asymmetric stretching modes. Perpendicular (<XXZZ>) 2D-IR spectrum of D_2O in DMF at c) T_w = 150 fs and d) T_w = 300 fs. Contour lines show signal intensities, orange and purple areas indicate spectral regions with positive and negative local Pearson correlation coefficients, respectively. Open blue symbols indicate the center-line position of the off-diagonal peak and the solid white line the center line.

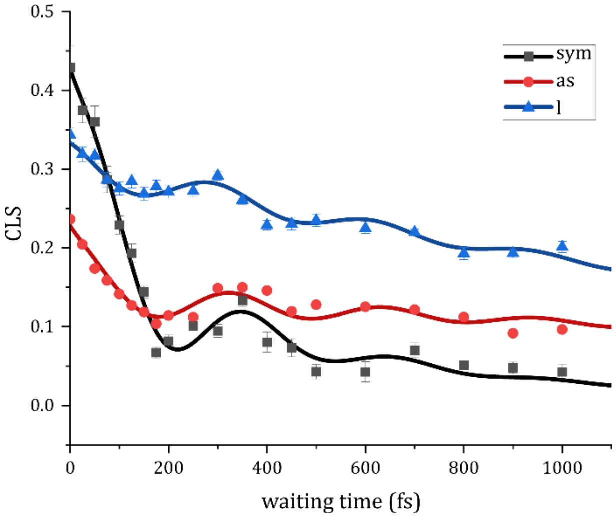

Supplementary Figure 6. CLS of the diagonal peaks of the symmetric (black), asymmetric (red), and local mode (blue). The solid lines are fits using Supplementary Eq. 6 with an additional exponential decay. For these fits the characteristic times τ_{osc}, τ_{damp}, and τ_{ex} are constrained to the values obtained from the cross-peak fit (Supplementary Table 7). The error bars are the standard error of the linear regression of the center line.

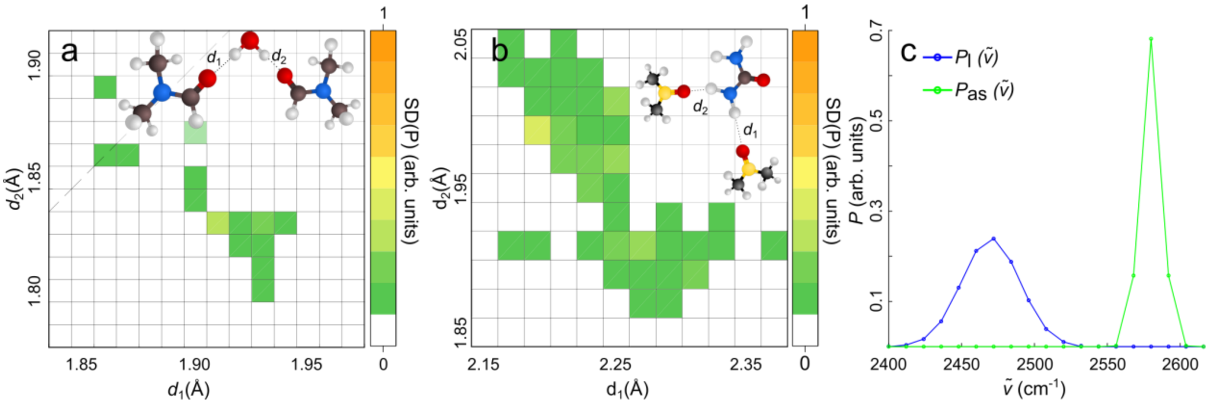

Supplementary Figure 7. Standard deviation of the population fit $P(d_1, d_2)$ for a) D_2O in DMF and b) urea-d4 in DMSO, obtained from ten random initial populations. c) Inhomogeneous frequency distributions $P_i(\tilde{\nu})$ for urea in DMSO. Symbols show discretized experimental values and solid lines show the fits based on the distribution in Figure 5c and the frequency maps in Supplementary Figure 11.

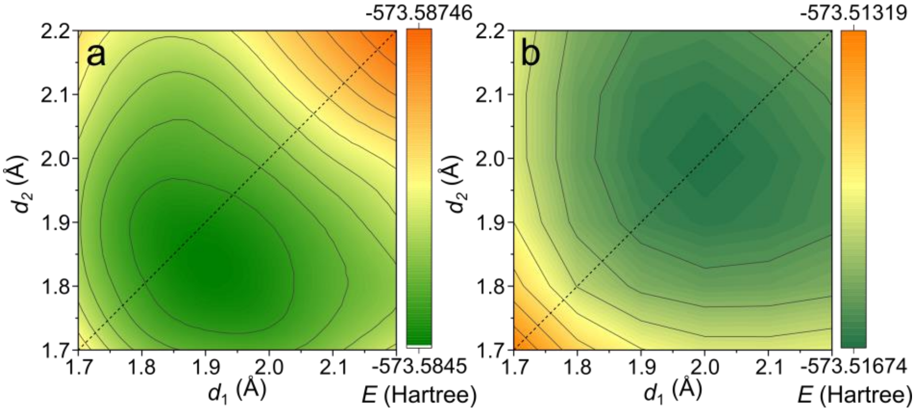

Supplementary Figure 8. Total energy of a D_2O molecule bound to two DMF molecules as a function of H-bond distances with the adjacent minimum energy reaction paths for H-bond breaking, in addition to the global minimum at the equilibrium distance of the H-bonds with D3 dispersion correction and conductor-like polarizable continuum model (CPCM) in a) and without dispersion correction and CPCM in b). Comparison suggests that the asymmetry of the energy profile in a) most likely stems from DMF-DMF interactions.

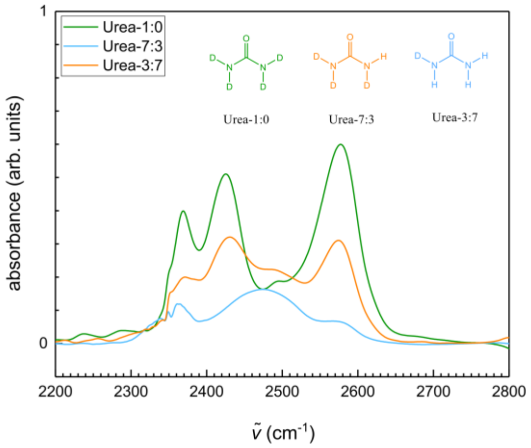

Supplementary Figure 9. Solvent subtracted IR spectra of urea-d4/urea mixtures in DMSO at different mixing ratios: pure urea-d4 (green), 70 % urea-d4 (orange), and 30 % urea-d4 (blue).

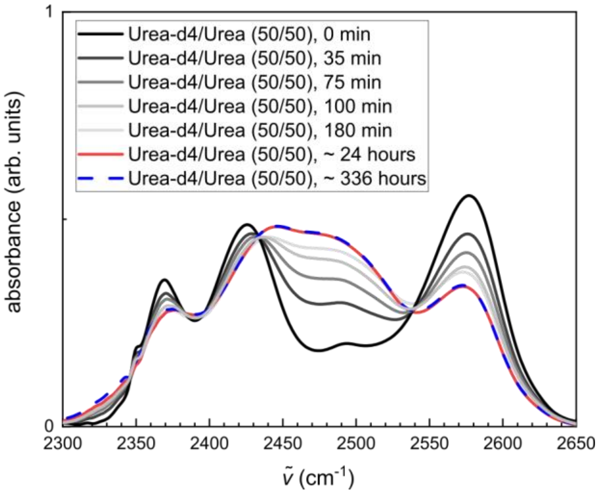

Supplementary Figure 10. Solvent-subtracted IR absorption spectra of a urea-d4/urea (50% / 50%) mixture in DMSO as a function of time after mixing at a concentration of c(urea) + c(urea-d4) = 0.36 mol/kg.

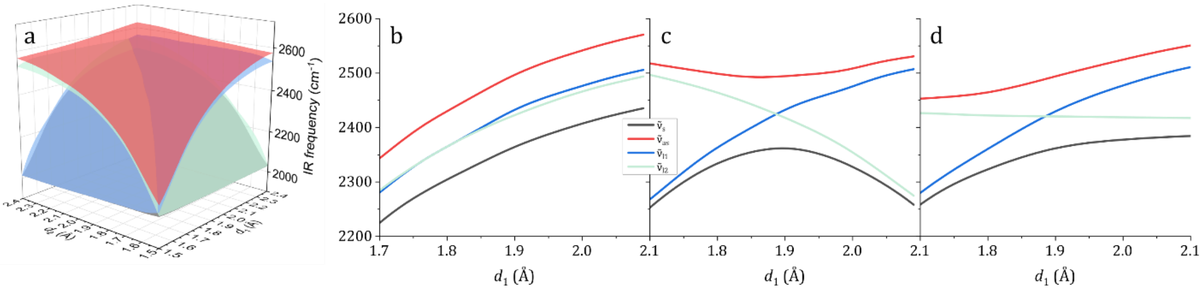

Supplementary Figure 11. a) Frequency maps for the symmetric $\tilde{\nu}_{sym}$(grey), local $\tilde{\nu}_{l1}$ (light blue), $\tilde{\nu}_{l2}$ (light green), and asymmetric $\tilde{\nu}_{as}$ (red) ND stretching modes of urea+4 DMSO as a function of hydrogen-bond distances d_1 and d_2, as obtained from the harmonic frequencies of relaxed surface scans using DFT calculations. b) Diagonal cut at $d_1 = d_2$ through the frequency maps representing correlated H-bonds, c) antidiagonal cut at $d_1 + d_2 = 3.8$ Å representing anti-correlated H-bonds, and d) cut at d_2 =1.9 Å representing uncorrelated H-bond distances.

SUPPLEMENTARY TABLES

Supplementary Table 1. Fitting parameters of the Gaussian fits for the linear spectra displayed in Supplementary Figure 1. D_2O in DMF was fitted with three Gaussians accounting for the overtone of the DOD bending mode (δ^2), the symmetric stretching (*sym*), and the asymmetric stretching (*as*).

	D_2O in DMF (δ^2)	D_2O in DMF (*sym*)	D_2O in DMF (*as*)	HOD in DMF (*l*)
Γ_G (cm^{-1})	143	79	78	94
$\tilde{\nu}_c$ (cm^{-1})	2414	2545	2640	2583
A_G	0.11	0.65	0.95	0.97

Supplementary Table 2. Parameters obtained from fitting Supplementary Eq. 1 to the antidiagonal cuts of the 2D-IR spectra of D_2O and HOD in DMF.

	D_2O (*sym*) antidiagonal at 2538 cm^{-1}	D_2O (*as*) antidiagonal at 2635 cm^{-1}	HOD (*l*) antidiagonal at 2552 cm^{-1}
$A_{\mathrm{ESA},i}$ (mOD)	0.13	0.10	0.03
$A_{\mathrm{GSB},i}$ (mOD)	-0.08	-0.17	-0.09
$\tilde{\nu}_{\mathrm{c,ESA},i}$ (cm^{-1})	2480	2600	2500
$\tilde{\nu}_{\mathrm{c,GSB},i}$ (cm^{-1})	2530	2630	2560
$\Gamma_{\mathrm{L,ESA},i}$ (cm^{-1})	104	47.1	63.0
$\Gamma_{\mathrm{L,GSB},i}$ (cm^{-1})	40.7	47.1	63.0

Supplementary Table 3. Fit parameters obtained from fitting Supplementary Eq. 2 to the diagonal slices of the 2D-IR spectra of D_2O and HOD in DMF.

	D_2O (*sym*) diagonal	D_2O (*as*) diagonal	HOD (*l*) diagonal
A_i (mOD)	-0.07	-0.15	-0.09
$\tilde{\nu}_{\mathrm{c},i}$ (cm^{-1})	2530	2630	2560
$\Gamma_{\mathrm{L,GSB},i}$ (cm^{-1})	40.7	47.1	63.0
$\Gamma_{\mathrm{G},i}$ (cm^{-1})	20.2	17.5	38.0

Supplementary Table 4: Parameters obtained from fitting Supplementary Eq. 1 to the antidiagonal slices of the 2D-IR spectra of urea in DMSO.

	urea-d4 (*sym*) antidiagonal at 2423 cm^{-1}	urea-d4 (*as*) antidiagonal at 2576 cm^{-1}	30% urea-d4 (*l*) antidiagonal at 2470 cm^{-1}
$A_{\mathrm{ESA},i}$ (mOD)	0.07	0.30	0.32
$A_{\mathrm{GSB},i}$ (mOD)	-0.22	-0.44	-0.57
$\tilde{\nu}_{\mathrm{c,ESA},i}$ (cm^{-1})	2390	2540	2420
$\tilde{\nu}_{\mathrm{c,GSB},i}$ (cm^{-1})	2420	2570	2470
$\Gamma_{\mathrm{L,GSB},i}$ (cm^{-1})	23.3	31.5	42.0

Supplementary Table 5: Parameters obtained from fitting Supplementary Eq. 2 to the antidiagonal slices of the 2D-IR spectra of urea in DMSO.

	urea-d4 (*sym*) diagonal	urea-d4 (*as*) diagonal	30% urea-d4 (*l*) diagonal
A_i (mOD)	-0.23	-0.46	-2.68
$\tilde{\nu}_{\mathrm{c},i}$ (cm^{-1})	2420	2580	2470
$\Gamma_{\mathrm{L,GSB},i}$ (cm^{-1})	23	31	42
$\Gamma_{\mathrm{G},i}$ (cm^{-1})	14.1	16.5	46.9

Supplementary Table 6: Signal decay times obtained from fitting Supplementary Eq. 4 to the integrated 2D-IR peaks.

	τ_{sym}(ps)	τ_{as}(ps)	τ_{cross}(ps)	τ_{l}(ps)
water	0.71 ± 0.06	0.59 ± 0.07	0.8 ± 0.1	0.8 ± 0.2
urea	0.83 ± 0.08	0.9 ± 0.2	1.0 ± 0.2	0.51 ± 0.02

Supplementary Table 7: Parameters obtained from fitting Supplementary Eq. 6 to the CLS data of the cross-peak of D_2O in DMF.

A_{osc}	τ_{osc} (fs)	ϕ	τ_{damp}(fs)	A_{ex}	τ_{ex}(fs)	CLS_{offset}
-0.059	310	0.98	470	0.049	50	0.021

SUPPLEMENTARY REFERENCES

1. Tokmakoff, A. & Fayer, M. D. Homogeneous vibrational dynamics and inhomogeneous broadening in glass-forming liquids: Infrared photon echo experiments from room temperature to 10 K. *J. Chem. Phys.* **103**, 2810–2826 (1995).

2. van der Post, S. T. *et al.* Strong frequency dependence of vibrational relaxation in bulk and surface water reveals sub-picosecond structural heterogeneity. *Nat. Commun.* **6**, 8384 (2015).

3. De Marco, L., Ramasesha, K. & Tokmakoff, A. Experimental Evidence of Fermi Resonances in Isotopically Dilute Water from Ultrafast Broadband IR Spectroscopy. *J. Phys. Chem. B* **117**, 15319–15327 (2013).

4. Ehrhard, A. A. *et al.* Elucidating Conformation and Hydrogen-Bonding Motifs of Reactive Thiourea Intermediates. *ACS Catal.* **12**, 12689–12700 (2022).

5. Tokmakoff, A. *et al.* Vibrational spectral diffusion and population dynamics in a glass-forming liquid: Variable bandwidth picosecond infrared spectroscopy. *J. Chem. Phys.* **102**, 3919–3931 (1995).

6. Bakker, H. J., Woutersen, S. & Nienhuys, H.-K. Reorientational motion and hydrogen-bond stretching dynamics in liquid water. *Chem. Phys.* **258**, 233–245 (2000).

7. Kandratsenka, A., Schroeder, J., Schwarzer, D. & Vikhrenko, V. S. Nonequilibrium molecular dynamics simulations of vibrational energy relaxation of HOD in D2O. *J. Chem. Phys.* **130**, 1–12 (2009).

8. Jansen, T. l. C., Cringus, D. & Pshenichnikov, M. S. Dissimilar Dynamics of Coupled Water Vibrations. *J. Phys. Chem. A* **113**, 6260–6265 (2009).

9. Saito, Y., Machida, K. & Uno, T. Infrared spectra of partially deuterated ureas. *Spectrochim. Acta Part A Mol. Spectrosc.* **27**, 991–1002 (1971).

10. Keuleers, R., Desseyn, H. O., Rousseau, B. & Van Alsenoy, C. Vibrational Analysis of Urea. *J. Phys. Chem. A* **103**, 4621–4630 (1999).
