# Supplementary material for: Dynamic anti-correlations of water hydrogen bonds
Source: Nat Commun. 2024 Dec 1;15:10453. doi: 10.1038/s41467-024-54804-y (PMC11609289; doi:10.1038/s41467-024-54804-y)
Supplement: Supplementary file 2 — Description of Additional Supplementary Files [file 41467_2024_54804_MOESM2_ESM.pdf]

Description of Additional Supplementary Files

File Name: Supplementary Data 1
Description: Optimized Coordinates of electronic structure calculations
